# Classification of regular and chaotic motions in Hamiltonian systems with deep learning

**DOI:** 10.1038/s41598-022-05696-9

**Published:** 2022-02-03

**Authors:** Alessandra Celletti, Catalin Gales, Victor Rodriguez-Fernandez, Massimiliano Vasile

**Affiliations:** 1 Department of Mathematics, University of Rome Tor Vergata, 00133 Rome, Italy; 2 Faculty of Mathematics, Al. I. Cuza University of Iaşi, 700506 Iaşi, Romania; 3 Department of Computer Systems Engineering, Universidad Politécnica de Madrid, 28031 Madrid, Spain; 4 Aerospace Centre of Excellence, Department of Mechanical and Aerospace Engineering, University of Strathclyde, Glasgow, G11XJ UK

**Keywords:** Applied mathematics, Computational science

## Abstract

This paper demonstrates the capabilities of convolutional neural networks (CNNs) at classifying types of motion starting from time series, without any prior knowledge of the underlying dynamics. The paper applies different forms of deep learning to problems of increasing complexity with the goal of testing the ability of different deep learning architectures at predicting the character of the dynamics by simply observing a time-ordered set of data. We will demonstrate that a properly trained CNN can correctly classify the types of motion on a given data set. We also demonstrate effective generalisation capabilities by using a CNN trained on one dynamic model to predict the character of the motion governed by another dynamic model. The ability to predict types of motion from observations is then verified on a model problem known as the forced pendulum and on a relevant problem in Celestial Mechanics where observational data can be used to predict the long-term evolution of the system.

## Introduction

The indicators to classify regular and chaotic motions are a powerful tool in dynamical systems, since they allow one to predict the character of a given trajectory. Even more ambitiously, one can extend the use of such dynamical indicators to investigate entire regions of the phase space. However, the calculation of most dynamic indicators requires a knowledge of the dynamic equations governing the system under investigation. On the other hand both in astronomy and satellite operations it is extremely valuable to predict the type of motion starting from observational data. This understanding can guide the development or update of a model or can be readily used to make operational decisions, in particular in the case of satellites in Earth orbit. Furthermore, a high resolution cartographic study of large portions of the phase space with dynamic indicators requires lengthy propagations of several trajectories starting from a large set of initial conditions. Thus, it would be desirable to reduce the cost and have faster ways to perform a large scale exploration of the phase space.

Deep Learning (DL) offers a potentially interesting solution to both problems as it could be used to classify types of motions from time series and would allow a more extensive cartographic study at a lower computational cost. However, the extent to which DL can be used to classify the type of motion in a general dynamical system and in particular in Celestial Mechanics is an open problem. This paper provides a partial answer. The contribution of this work is to assess the effectiveness of some families of state-of-the-art DL methods, when trained with classical dynamical indicators, at classifying types of motion from time series. The paper will demonstrate also the generalization capabilities of InceptionTime deep Convolutional Neural Networks (CNN) that would allow the cartographic study of analogous albeit not identical dynamical systems. A review of recent machine learning (ML) methods used in dynamical astronomy can be found in^1^, while some advanced techniques to learn dynamical systems can be found in^2^. Our work differentiates from the recent literature on ML for dynamical systems and dynamical astronomy in that we provide some evidence of the ability of DL at classifying types of motion from dynamic indicators associated to time series. Our results complement and extend recent findings on DL for the classification of chaotic time series^3^.

In this work, we considered a set of conservative models with increasing complexity. We started with a pendulum described by a one-dimensional, time-dependent Hamiltonian function (hence depending upon an action *y*, an angle *x* and time *t*) with a potential composed by two harmonics. Next, we considered a modified pendulum with the potential given by three and then by four harmonics. In all pendulum-like models, we analyzed the phase space using dynamical indicators to detect three kinds of motion: chaotic, rotational, librational, where the latter two motions are characterized by an amplitude of variation of $x(2 n \pi )$, $n\in {\mathbb {N}}$, within the whole range $0^{\circ }$–$360^{\circ }$ (rotational motion) or a subset of the interval $0^{\circ }$–$360^{\circ }$ (librational motion). In view of training a DL model with a classification method based on dynamical indicators, we compared different techniques: Fast Lyapunov Indicators (FLIs) and frequency map analysis (FMA), in the latter case using two different approaches to compute the dynamical index that provides the information on the chaotic, rotational, librational character of the motion.

Given an initial condition, we generated a finite number of random time series as a solution at different intervals of time with fixed step size. For each time series we computed the dynamical indicators to which we associate indexes to distinguish between chaotic, rotational and librational motions. Then, we trained an InceptionTime CNN^4^ with a set of indexes and corresponding time series. InceptionTime, which has been extensively used to classify time series data^5^ is composed by five deep CNN models, each one being formed by cascading multiple Inception modules^6^, with the property to have the same architecture, but different values for the initial weights. In order to validate our choice of DL method we performed also a comparison among different DL architectures, which showed the greater accuracy of InceptionTime and, in general, of CNN-based networks. In recent times a number of authors have applied Machine Learning (ML) to the study of dynamical systems, developed dynamics-informed ML algorithms or used ML to discover the underlying dynamics from data^7–11^. In the specific framework of Celestial Mechanics ML algorithms have been used to analyze the solution of the 3-body problem^12^, the identification of asteroid families through proper elements^13^ or the re-discovery of Kepler’s laws^14^. Although the use of ML in the analysis of physical systems has to be considered with care^15^, we will demonstrate that Deep Learning for the classification of time series in dynamical systems can offer a powerful tool to identify types of motion in a specific problem of Celestial Mechanics.

As a case study, we will apply Deep Learning to the analysis of the spin–orbit problem, which describes the rotational dynamics of a satellite moving around a central body and whose center of mass orbits on a Keplerian ellipse. Under some simplifying assumptions, the Hamiltonian of the spin–orbit problem has a pendulum-like structure, but with a potential that has an infinite number of harmonics with coefficients that decay with powers of the orbital eccentricity. When the eccentricity is small, it is convenient to approximate the potential with a trigonometric series. Like in the pendulum case, we trained an InceptionTime CNN with time series generated from a potential given by a few harmonics and then we classify the dynamics associated to a model with the potential approximated by a larger number of harmonics.

The paper is structured as follows. In the first part, after a presentation of the dynamic models, we will introduce the dynamic indicators used in the classification of chaotic and regular motions. In the second part of the paper we will introduce the Deep Learning algorithms, their training with the dynamic indicators and their application to the classification of motion in the dynamical systems presented in the first part. We will extensively test different architectures and learning models with a combination of data to assess which one provides the more reliable classification of the dynamics. We will conclude the paper with a discussion on the applicability of Deep Learning in Celestial Mechanics starting from the evidence collected in this work.

## Dynamic models: the forced pendulum and the spin–orbit problem

We consider two different models widely studied in the literature, the forced pendulum and the spin–orbit problem in Celestial Mechanics. Such models are described by a one-dimensional, time-dependent, nearly-integrable Hamiltonian of the form

$$\begin{aligned} H(y,x,t)=h(y)+\varepsilon f(y,x,t)\ , \end{aligned}$$

where $y\in Y$, *Y* an open set of ${{\mathbb {R}}}$, $(x,t)\in {{\mathbb {T}}}^2$ with (*y*, *x*) conjugated action-angle variables; the *integrable *part *h* and the *perturbing *function *f* are assumed to be analytic functions, while $\varepsilon >0$ is the *perturbing parameter. *For $\varepsilon =0$ the Hamiltonian reduces to the integrable case $H(y,x)=h(y)$; denoting by $\omega (y)= \frac{\partial h(y)}{\partial x}$ the *frequency *or *rotation number, *and denoting by (*y*(0), *x*(0)) the initial conditions, then Hamilton’s equations can be integrated as $y(t)=y(0)$, $x(t)=x(0)+\omega (y(0))\, t$.

### The forced pendulum

For $(y,x)\in {{\mathbb {R}}}\times {{\mathbb {T}}}$ and for $\varepsilon \in {{\mathbb {R}}}_+$, the *forced pendulum *is described by the Hamiltonian system:

1 $$\begin{aligned} H_2(y,x,t)={y^2\over 2}-\varepsilon \ \Big (\cos (x)+\cos (x-t)\Big )\ . \end{aligned}$$

**Figure 1:**
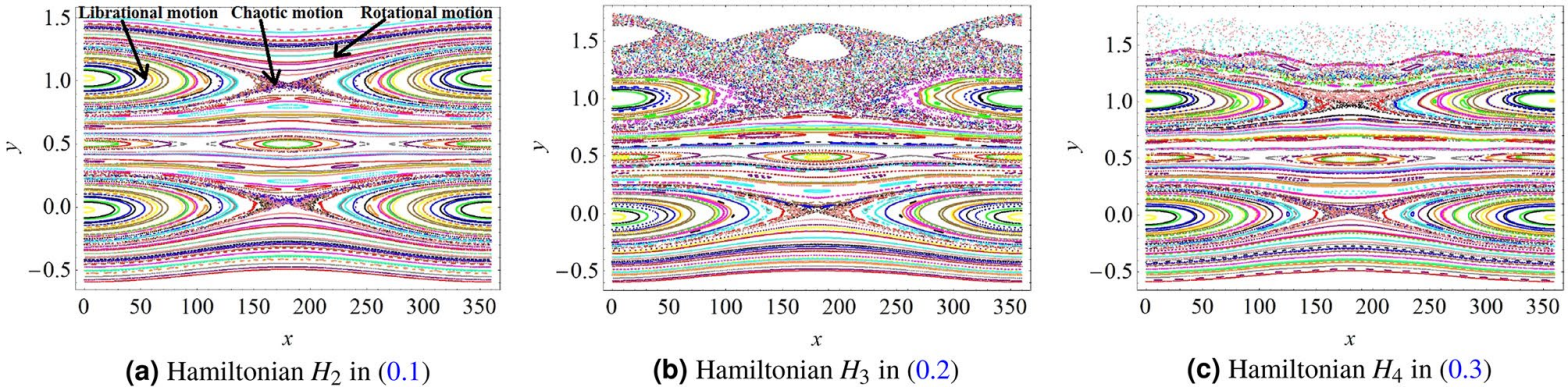
Poincaré maps associated to Hamiltonians (1), (2) and (3) for $\varepsilon =0.02$, $5 \times 10^5$ iterations and a time step equal to $h={{2\pi }\over {10^3}}$.

In the integrable case, the frequency is given by $\omega (y)=y$; the motions are periodic, whenever $\omega$ is a rational number, and quasi-periodic if $\omega$ is irrational. For $\varepsilon \not =0$, Hamilton’s equations associated to (1) are non-integrable, and display regular and chaotic motions. Such motions are conveniently seen when computing the *Poincaré map, *which consists in plotting the intersection of the solution with the plane $t=0$ mod $2\pi$. Figure 1a shows the Poincaré map for $\varepsilon =0.02$, for a grid of $12 \times 12$ initial conditions with the variable *y* varying between $-$0.5 and 1.5 and *x* ranging from $0^{\circ }$ to $180^{\circ }$. In the remainder of the paper, solutions are obtained by numerical integration of the equations of motion, derived from each of the Hamiltonians, through a 4-th order Runge–Kutta method.

The motions can be either *librational,* *rotational *and *chaotic, *the latter being characterized by an extreme sensitivity to the choice of the initial conditions. Figure 1a highlights typical examples from each class of motions. In the following we will train different DL models using series of values $(x(t_j),y(t_j))$ taken at times $t_0=0, t_1=2 \pi ,...,t_1=2 j\pi ,...,t_N=2N\pi$, for a suitable $N\in {{\mathbb {N}}}$. The choice of this particular time series is motivated by the need of characterizing all types of librational motion in terms of the angular variable *x*. Indeed, the class of librational motions considered here includes all possible resonant motions (see the libration islands depicted in Fig. 1); namely, the motions for which there exists an angular variable of the form $\varphi _{nm}=n x-m t$ mod $2 \pi$, $n, m\in {\mathbb {Z}}$, that takes values within a subset of the interval $[0, 2\pi )$. The Poincaré map provides a complete characterization of the dynamical system, even if the resonant angles are different. On the other hand, in many real applications one is usually interested in a single type of librational motion (a specific resonance), which involves one critical angle. For such cases, the property of a resonant angle librating within a subset of the interval $[0, 2\pi )$ can equally be described by time series obtained at random time steps.

### The modified forced pendulum

On top of the forced pendulum in (1), we consider two modified versions obtained by adding, respectively, one and two trigonometric components to the Hamiltonian (1). This leads to the following Hamiltonians:

2 $$\begin{aligned} HH_3(y,x,t)= & {} {y^2\over 2}-\varepsilon \ \Big (\cos (x)+\cos (x-t)+{1\over 2}\cos (2x-3t)\Big ) \end{aligned}$$

3 $$\begin{aligned} H_4(y,x,t)= & {} {y^2\over 2}-\varepsilon \ \Big (\cos (x)+{4\over 5}\cos (x-t)+{1\over {10}}\cos (2x-3t)+{1\over 5}\cos (5x-8t)\Big )\ . \end{aligned}$$

The motivation for introducing the Hamiltonians (2) and (3) is to assess how DL can deal with types of motion characterized by more complex time series. In particular we are interested in testing the ability of DL algorithms to classify the time series coming from one Hamiltonian after being trained on the time series coming from another Hamiltonian. Despite the similarities of the Hamiltonians in (1)–(2)–(3), it is well known that these systems can exhibit substanitally different behaviors with respect, for example, to boundaries of analyticity and renormalization properties^16^. The Poincaré maps associated to (2) and (3) are shown respectively in Fig. 1b, c.

### The spin–orbit problem

In this section we introduce the *spin–orbit problem *in Celestial Mechanics, which consists in the study of the motion of a rigid satellite, say *S* of mass *m*, with triaxial structure, subject to the gravitational attraction of a point–mass planet, say *P* with mass *M*. We make the following assumptions:

(i) the satellite *S* orbits on a Keplerian ellipse with semimajor axis *a* and eccentricity *e*;

(ii) the spin–axis is aligned with the smallest physical axis of the satellite;

(iii) the spin–axis is perpendicular to the orbital plane.

**Figure 2:**
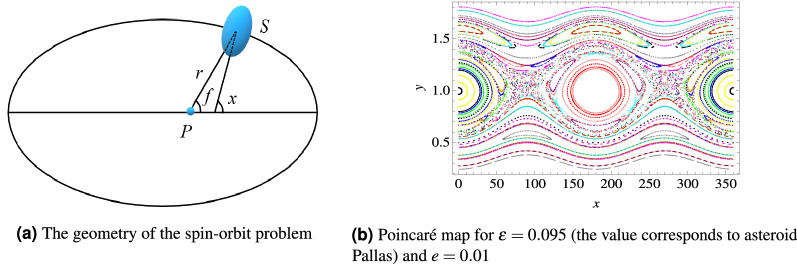
The spin–orbit problem.

We adopt the units of measure such that the mean motion, which by Kepler’s law coincides with $\sqrt{{{{\mathscr {G}}}}M/a^3}$, with ${{{\mathscr {G}}}}$ the gravitational constant, is equal to one. We define the parameter $\varepsilon \equiv {3\over 2}{{I_2-I_1}\over I_3}$, related to the moments of inertia $I_1 \le I_2 \le I_3$ of the satellite. Next, we introduce the angle *x* formed by the direction of the largest physical axis (which by assumptions (ii) and (iii) belongs to the orbital plane) with the periaxis direction (see Fig. 2a). Then, the Hamiltonian takes the form^17^

4 $$\begin{aligned} H(y,x,t)={{y^2}\over {2}}-{\varepsilon \over 2}\left( {a\over r}\right) ^3 \cos (2x-2f)\ , \end{aligned}$$

where *r* denotes the orbital radius and *f* is the true anomaly of the Keplerian ellipse. A Poincaré map for the Hamiltonian (4) is shown in the right panel of Fig. 2b.

Note that the parameter $\varepsilon$ vanishes for equatorial symmetry, namely $I_1=I_2$; correspondingly, the Hamiltonian (4) becomes integrable. Due to assumption (i), the orbital radius and the true anomaly are known functions of the time, being determined through the Keplerian relations:

5 $$\begin{aligned} r= a(1-e\cos u)\ ,\qquad f=2\arctan \Big (\sqrt{{1+e}\over {1-e}}\tan {u\over 2}\Big )\ , \end{aligned}$$

where *u* is related to the mean anomaly $\ell _0$ by means of Kepler’s equation $\ell _0=u-e\sin u$. According to the relation (5), *r* and *f* depend on the eccentricity *e*; for $e=0$ one has $r=a$, $f=t+t_0$ for a suitable constant $t_0$ and the Hamiltonian (4) becomes integrable. Expanding (5) in power series of the orbital eccentricity, the Hamiltonian (4) can be developed in Fourier series as in^17^; retaining the coefficients up to order 4 in the eccentricity, we obtain the following Hamiltonian:

6 $$\begin{aligned} H_{SO}(y,x,t)= & {} {{y^2}\over {2}}-\varepsilon \Big [\left( -{e\over 4}+{e^3\over {32}}\right) \cos (2x-t)+\left( {1\over 2}-{5\over 4}e^2+{{13}\over {32}}e^4\right) \cos (2x-2t)+\left( {7\over 4}e-{{123}\over {32}}e^3\right) \cos (2x-3t)\nonumber \\&\quad +\left( {{17}\over 4}e^2-{{115}\over {12}}e^4\right) \cos (2x-4t)+{{845}\over {96}}e^3\cos (2x-5t)+ {{533}\over {32}}e^4\cos (2x-6t)\Big ]\ . \end{aligned}$$

## Indexing methods for regular and chaotic motion

The regular and chaotic behavior of the dynamic models (1)–(2)–(3) and (6) can be numerically assessed with chaos indicators. The idea is to use chaos indicators to associate the index 0, 1 or 2 to, respectively, a chaotic, librational or rotational type of motion. We will then train a DL model to automatically associate the same index values to time series. The goal is to train the DL algorithm so that given a time series $(x(t_{j_k}),y(t_{j_k}))$, starting from some initial condition (*x*(0), *y*(0)), the DL can automatically identify if the time series corresponds to a chaotic, librational or rotational motion.

Among the possible chaos indicators we selected the Fast Lyapunov Indicators (hereafter FLI^18^) and the frequency map analysis^19^ (see references^20^ and^21^ for alternative chaos indicators). In this section we present these two indicators and the approach to index the time series. As detailed below, both indexing methods involve an empirical choice of some threshold parameters. The value of the thresholds is manually tuned by comparing the resulting index against the Poincaré map. For this reason, as it will be explained in this section, each indexing method has some limitations and can produce indexing errors that have an impact on the training of the DL and its ability to predict the right type of motion. In the section on deep learning classification we will show the effect of indexing errors and how each indexing method performs.

### Fast Lyapunov indicators

Given the vector field $X=X(z,t)$ for $z\in {{\mathbb {R}}}^n$, $t\in {{\mathbb {R}}}$, we write the equations of motion and the associated variational equations as

$$\begin{aligned} \frac{d z}{d t}=X(z, t)\ , \qquad \frac{d v}{d t}=\frac{\partial X}{\partial z}(z, t)v\ . \end{aligned}$$

Let (*z*(0), *v*(0)) be an initial conditions with $|\!|v(0)|\!|=1$; then, the FLI at time $t=T$ is defined as

$$\begin{aligned} \mathrm{FLI}(z(0), T) \equiv \sup _{v(0) \in {\mathbb {R}}^n,|\!|v(0)|\!|=1}\ \sup _{0 < t\le T} \log \Vert v(t)\Vert \ . \end{aligned}$$

Each initial condition (*x*(0), *y*(0)) is integrated for a time span of $T=400 \pi$ with a step size $h=2 \pi /10^3$; the integration produces a time series $(x(t_j),y(t_j))$ with $j=0,...,N$ and $N=T/h=2\times 10^5$. For DL classification, we consider a subsequence $(x(t_{j_k}),y(t_{j_k}))$ of the time series $(x(t_j),y(t_j))$, obtained by taking $j_k=10^3 k$, $k \in {\mathbb {N}}$, $k \le 200$. This corresponds to retaining just the terms for which $t=0\ {\text {mod}}\ 2\pi$, similarly to the Poincaré map.

**Figure 3:**
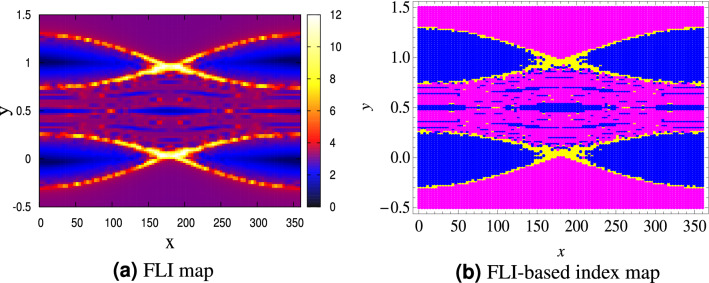
FLI map and FLI-based index map of the dynamics index in the case of the dynamic model (1) for $\varepsilon =0.02$. The colors are coded as follows: yellow—chaos, magenta—rotational motions, blue—librational motion.

Given a dynamical model, for each value of the parameter $\varepsilon$, we compute the FLI for a grid of $101\times 101$ equally spaced points of the *x*–*y* plane, where *x* ranges in the interval $[0^{\circ }, 360^{\circ })$, while the action *y* spans the interval $[-0.5, 1.5]$. Figure 3a shows the FLI values through a color scale for Hamiltonian (1), where $\varepsilon =0.02$. Lower values of the FLIs, corresponding to darker color regions in Fig. 3a, represent regular motion, either periodic or quasi–periodic, while higher values of he FLIs, corresponding to lighter color regions in Fig. 3a, represent chaotic motions.

In order to assign an index to each initial condition we first define two threshold values $FLI_{l}$ and $FLI_{h}$ such that: for $FLI>FLI_{h}$ the initial condition corresponds to a chaotic motion and the index is 0, for $FLI_{l}\le FLI\le FLI_{h}$ the initial condition corresponds to a rotational motion and the index is 2, for $FLI_{l}\ge FLI$ the initial condition corresponds to a librational motion and the index is 1. Note that there is no analytical criterion that can be used to define the threshold values $FLI_{l}$ and $FLI_{h}$, thus they need to be chosen empirically, by comparison with the corresponding Poincaré map. In the following we use the fixed values $FLI_h=3.9$ and $FLI_l=3$. Finally, since the real dynamics is complex, using only three classes might not be sufficient to index all forms of motion. Depending on the value of $\varepsilon$, some small libration islands, which account for secondary resonances, could appear inside the libration regions corresponding to the main resonances. In the remainder of the paper secondary resonances will still be classified as librational. This will represent a challenge for the DL because time series displaying different patterns will receive the same index. We also expect that inside the libration regions and close to the separatrix there will be possibly librational motions, mislabelled as rotational. Figure 3b shows an example of dynamics index map for dynamic model (1) with $\varepsilon =0.02$.

### Frequency map analysis

The global dynamics of a dynamical system can be conveniently studied by looking at the behavior of its frequency. According to KAM theory^22^, invariant tori for (1) exist provided the Kolmogorov non-degeneracy condition is satisfied (namely the determinant of the Hessian matrix of the integrable part is non-zero), implying that the frequency map $\omega :Y\rightarrow {{\mathbb {R}}}$ is a local diffeomorphism. With reference to the forced pendulum (1) and denoting the time series starting from (*x*(0), *y*(0)) with $(x(t_j),y(t_j))$, $j=0,1,...$, one can define the frequency (or rotation number) of a dynamical system as

7 $$\begin{aligned} \omega =\lim _{n \rightarrow \infty } a_n, \qquad \quad a_n= {1\over n}\ \sum _{j=1}^n y(t_j)\ . \end{aligned}$$

**Figure 4:**
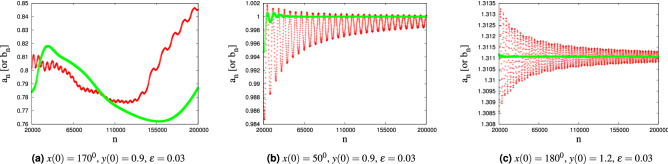
The terms of the series $a_n$ (red) and $b_n$ (green), $2\times 10^4\le n\le 2 \times 10^5$, for chaotic (**a**), librational (**b**), and rotational (**c**) motions, for the Hamiltonian (1).

When the limit exists, if $\omega$ is a rational number then the motion is periodic, while if $\omega$ is an irrational number then the motion is quasi-periodic. Figure 4 shows the terms of the series $a_n$ with $2\times 10^4\le n \le 2\times 10^5$ (red curves) for three types of motion: chaotic, librational and rotational. The oscillations of the red curves in Fig. 4b, c show that a large numbers of terms of the series $a_n$ should be computed to reach enough accuracy.

The rotation number can be computed in a more efficient way by using the weighted Birkhoff averages^23^ with the frequency computed as

8 $$\begin{aligned} \omega = \lim _{n \rightarrow \infty } b_n, \qquad \quad b_n ={1\over {\sum _{j = 1}^n \phi (\tfrac{j}{n})}}\ \sum _{j = 1}^n y(t_j) \phi (\tfrac{j}{n}) \quad \mathrm{with}\ \phi (t)= \left\{ \begin{array}{ll} \exp \biggl (\frac{-1}{t(1-t)} \biggr )\,, &{} t \in (0,1) \\ 0\,, &{} t\le 0 \quad {\text {or}} \quad t \ge 1\ .\\ \end{array} \right. \end{aligned}$$

**Figure 5:**
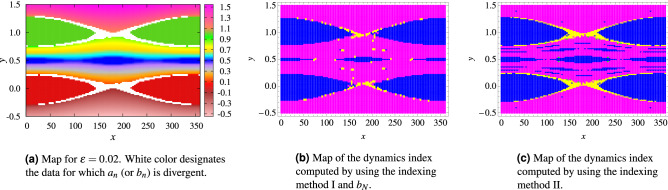
Frequency-based maps for the dynamic model (1), where $\varepsilon =0.02$. The colors for (**b**) and (**c**) are coded as follows: yellow—chaos, magenta—rotational motions, blue—librational motion.

The green curves in Fig. 4 correspond to the series $b_n$, $2\times 10^4\le n \le 2\times 10^5$; when the rotation number exists, the method of weighted Birkhoff averages provides more accurate results than the classical approach and it is converging (within a given precision) on a shorter time span. Considering a dynamic model and fixing a value of $\varepsilon$, we compute the rotation number (if it exists) for a grid of $101\times 101$ equally spaced points of the *x*–*y* plane, where *x* ranges in the interval $[0^{\circ }, 360^{\circ })$, while the action *y* spans the interval $[-0.5, 1.5]$. Figure 5a shows a color map of the values of $\omega$ for Hamiltonian (1), with $\varepsilon =0.02$. White color indicates the initial conditions for which $a_n$ or $b_n$ is divergent. In the following, we will use two methods to compute a rotation-number-based dynamics index with the frequency computed either through (7) or (8): the first approach uses the monotonicity property of the frequency, while the second method uses the convergence property of the sequences $a_n$ or $b_n$.

#### Indexing method I: monotonicity

We use the monotone character of the rotation number in the following way. For a given value of $\varepsilon$ and for a fixed value of *x*(0), we compute the frequency as a function of the initial condition *y*(0) and determine the graph of $a_N$ (or $b_N$) versus *y*(0). The behavior of the graph allows us to characterize the dynamics as follows: a monotone increase of $a_N=a_N(y)$ (or $b_N=b_N(y)$) corresponds to a region of invariant rotational tori; a plateau of the graph denotes a librational region; an irregular behavior of $a_N=a_N(y)$ (or $b_N=b_N(y)$) corresponds to a chaotic region. This leads us to associate a dynamics index to each initial condition *y*(0). Precisely, given $y_1(0)< y_2(0)< y_3(0)$, if $a_N(y_1(0)) \le a_N(y_2(0)) \le a_N(y_3(0))$ then the motion corresponding to $(x(0), y_2(0))$ is marked as regular. If, in addition, $|a_N(y_2(0))-a_N(y_1(0))|\le \delta$ and $|a_N(y_3(0))-a_N(y_2(0))|\le \delta$, where $\delta$ is a small number measuring the precision of the computation (see Fig. 4), then the motion is labeled as librational, otherwise the motion is labeled as rotational. If the condition $a_N(y_1(0)) \le a_N(y_2(0)) \le a_N(y_3(0))$ is violated, then the motion is labeled as chaotic. A similar procedure is used with the sequence $b_N$. Figure 5b shows the map of the dynamics index derived by using $b_N$ for (1) and $\varepsilon =0.02$. The main source of indexing error, in this case, comes from the choice of an optimal value for $\delta$ and the limitations to detect tiny resonant (or chaotic) regions.

#### Indexing method II: convergence

In case of convergence, the frequency can be accurately estimated by $b_N$; in particular, if $b_N$ is a rational number then the motion is labeled as librational, while if $b_N$ is an irrational number then the motion is labeled as rotational. The dynamics index is computed as follows. We consider the terms $b_n$ with $N/3\le n\le N$; when the difference between the smallest and largest terms of such sub-sequence is one order of magnitude larger than the accuracy with which the frequency is computed, then the motion is labeled as chaotic. On the contrary, in the case of convergence, we use the numerical method described in^23^ for establishing whether the estimated frequency $\omega$ is a rational or an irrational number, which consists in finding the rational number *p*/*q*, $p \in {\mathbb {Z}}$, $q \in {\mathbb {N}}$ with the smallest *q* in the interval $(\omega -\delta , \omega +\delta )$. Figure 5c shows the maps of the dynamics index for (1) and $\varepsilon =0.02$. The main source of indexing error, in this case, is establishing the threshold value for discriminating between chaotic and regular motions.

## Deep learning classification

This section describes which deep learning algorithm we chose, how it was trained to classify the motion governed by dynamic models (1)–(2)–(3) and (6) and how it compares to other DL algorithms. After a number of experiments with different DL algorithms we found that InceptionTime^4^, an architecture based on Convolutional Neural Networks (CNN), performed particularly well at classifying the time series under investigation in this paper. InceptionTime applies, at each layer of a CNN, a set of convolution operations to the time series. Each convolution operation creates a new transformation of the input data (also known as *feature maps*), so that the series become easier to be classified by the CNN.

The library tsai^24^, based on the deep learning library *fastai*^25^, was employed for the implementation (the source code will be accessible in https://github.com/vrodriguezf/mlchaos). For all experiments the network was trained for 10 epochs with batch size of 512 elements, a one-cycle learning rate schedule with its maximum learning rate set up as suggested in^26^, and the *Lookahead* optimizer^10^ (also known as RAdam), with a momentum of 0.95 and a weight decay of 0.01. The rest of the hyperparameters was set as default from the aforementioned libraries. No normalization was applied to the input data.

Since the goal is to classify time series, the loss function we selected is the cross-entropy loss, in which one first takes the softmax activation function of the model output to ensure that the activations are all between 0 and 1 and sum to 1, and then takes the log-likelihood of that with respect to the target label^25^. Further, we added label smoothing^6^ in the loss to prevent the network from predicting overconfidently. This is especially important since the data may not be indexed very accurately with the indexing methods described in previous sections. In the remainder of this section we present the results of a number of experiments aimed at assessing the performance of InceptionTime including an analysis of the effect of different indexing methods, a comparative analysis against other DL architectures, an analysis of the generalization capabilities of the DL method, an analysis of the effect of the number of samples in the time series and finally an analysis of the applicability of DL to the spin–orbit problem.

### Comparison among indexing methods

**Figure 6:**
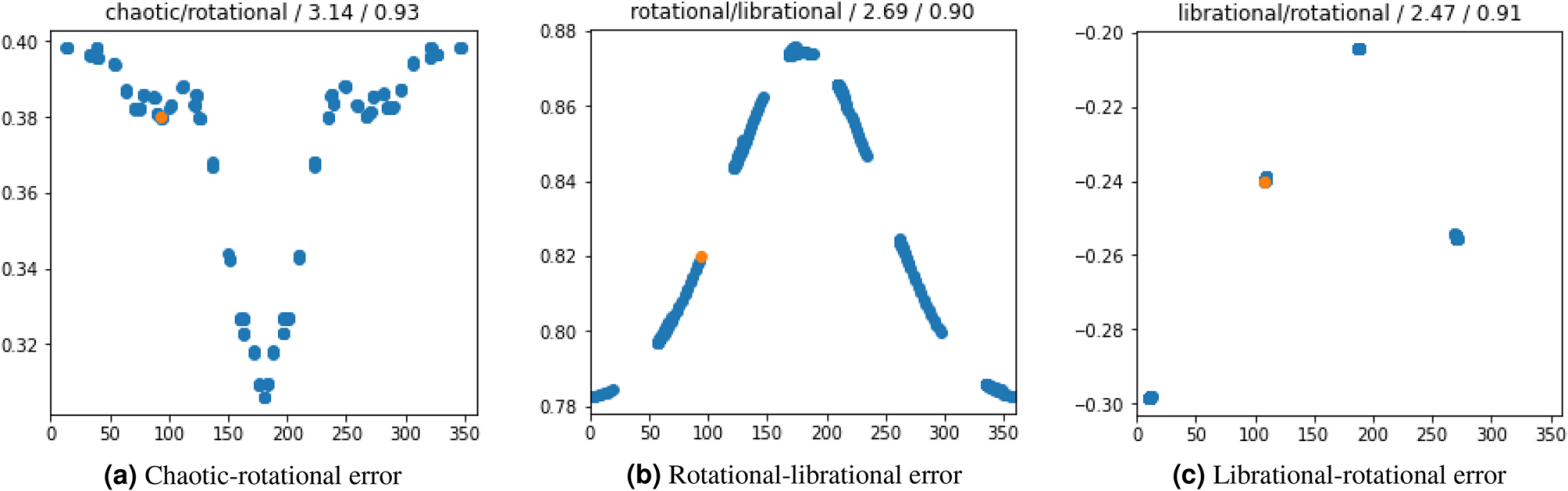
Motions with largest errors (values of the loss function) from the validation set of the first experiment, in which the network trained with data associated to the forced pendulum (1), with $\varepsilon =10^{-2}$, $3\times 10^{-2}$, and $5\times 10^{-2}$, labelled using the FLI, and validated on a random subset of the training data. The initial point of the motion is marked in orange. Annotations on top of each image represent: DL prediction/FL index/value of the loss function/probability of the predicted label.

We started by training the InceptionTime on a set of time series generated with the forced pendulum in (1), for $\varepsilon =10^{-2}$, $3\times 10^{-2}$, $5\times 10^{-2}$. For each value of $\varepsilon$, 10,201 time series, corresponding to 101 $\times$ 101 initial conditions $(x_0, y_0)$, were created. Each time series is numerically computed by integrating the canonical equations of motion, through a 4-th order Runge–Kutta algorithm, for a time span of $T=400 \pi$ with a step size $h=2 \pi /10^3$. The DL input data are produced by considering a sub-sequence $(x(t_{j_k}),y(t_{j_k}))$ of the generated time series $(x(t_j),y(t_j))$ with $j=0,...,N$ and $N=T/h=2\times 10^5$, obtained by taking $j_k=10^3 k$, $k \in {\mathbb {N}}$, $k \le 200$. This means that each time series is characterized by 200 points in the (*x*, *y*) plane. A total of $80\%$ of the time series, chosen at random from the dataset, were used for training, while $20\%$ were used for validation, to ensure that the network was not overfitting to the training data. The Fast Lyapunov Indicator (FLI) was used to index each time series. With this setup, the validation achieved an accuracy of $97\%$. Although this is a very good accuracy we investigated where the loss of accuracy was coming from. Figure 6, shows the Poincare’ section of some of the motions from the validation set for which the network made wrong predictions with high confidence, large values of the loss function, with respect to the FLI indexing. Figure 6a is a chaotic motion that the DL correctly identified as chaotic but the indexing labelled as rotational. Figure 6b is a librational motion that the indexing method correctly labelled but the DL incorrectly identified as rotational and finally Fig. 6c is a librational motion that the indexing method incorrectly labelled as rotational. The orange dot is the initial condition.

This observation demonstrates that an inaccurate indexing can lead to an incorrect validation because the DL can learn the right pattern even when the index is wrong. At the same time an accurate indexing is key to differentiate borderline cases that are otherwise difficult to predict. In order to better understand the influence of the indexing method we performed further experiments to compare the use of FLI and the frequency map analysis with both the monotonicity and convergence methods.

**Figure 7:**
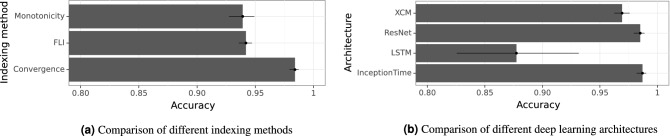
Performance comparisons for model (1).

We used 5 datasets of time series generated from (1) with $\epsilon =0.021,0.022,0.023,0.024,0.025$ respectively. For each indexing method, we train the network 5 times, leaving out each time one of the datasets for validation and using 4 left for training. In all cases, we used InceptionTime as deep learning architecture. The results of the experiment are shown in Fig. 7a. As it can be seen, the convergence method leads to the best validation performance. Therefore, we used this indexing method for the rest of the experiments. It is worth mentioning that we also tried using all indices together instead of choosing one of them at the time, but this did not increase the accuracy of the prediction with InceptionTime.

### Comparison among deep learning architectures

In order to pick InceptionTime as our DL method of choice, we compared the following deep learning architectures for time series classification.

- *InceptionTime*: It has become one of the most popular for the task of time series classification. It employs one single block 6 “inception modules”, which allows to use multiple types of filter size, and adds residual connections every 3 modules. The detailed configuration of each module (number of filters, filter size, bottleneck, etc.) can be seen in the original paper^4^.
- *ResNet*: proposed in^27^, this is a 1-dimensional adaptation of the popular ResNet for images that revolutionized computer vision with the inclusion of residual connections that allow training deeper convolutional neural networks. We employ a network with 3 residual blocks, each of them made up of 3 convolutional layers with filter sizes 7, 5, and 3 respectively. The number of filters created within each block is kept constant, but varies across blocks, being 64 for the first one and 128 for the two and the third.
- *XCM*: An explainable convolutional neural network^28^ for time series classification. It comprises two parallel convolutional blocks of 2 layers each, with 128 filters per layer. One of the blocks employs normal 1-dimensional convolutions on the time dimension, while the other does 2D convolutions on both the time and the variable dimensions.
- *LSTM*: Long Short Term Memory (LSTM) networks are a type of Recurrent Neural Networks, where the activations of the network are laid out as a loop, which allows for a better learning of sequential data. We employ a network with 1 hidden layer and 100 LSTM cells in it. There is no bidirectionality, as the network takes the input only in a forward direction.

All of the networks have been trained with the optimizer, batch size, number of epochs and learning rate configured as detailed at the beginning of this section, and all of them use ReLU as activation function. The reason why we chose these architectures and not others for the comparison is because they are good representatives of the two biggest families of neural networks to process sequences: CNNs (both 1-dimensional, 2-dimensional and their extensions with residual connections) and RNNs, represented by the LSTM, which is by far the most used architecture of this type. To be noted that specific developments within each of these families could produce better results than the one in this. However, benchmarking deep learning algorithms is out of the scope of this paper. We deem the comparison in this section sufficient to draw reasonable conclusions on the applicability of these families of DL architectures to the type of problems covered in this paper. We leave for future work the use of the Transformer architecture^29^, which has recently become the state of the art in many other domains and tasks supported by deep learning.

We present only the results with the best performing indexing method, the convergence method. Analogous results were obtained also with the other indexing methods. Figure 7b shows that CNN-based networks (Resnet, XCM, InceptionTime) outperform other architectures such as LSTM. Furthermore, InceptionTime outperforms all the other architectures with a 99% average accuracy. This is inline with the current literature, where InceptionTime stands out among the rest of deep learning architectures for multivariate time series classification^5^.

### Generalization capabilities

We assessed the generalization capabilities of InceptionTime by performing three types of experiments: first we trained the network on a dynamic model and then used it to predict the time series of a different dynamic model; second we trained the network on a reduced set of initial conditions and we tried to complete a cartography within the same range of values of the initial conditions used for training; finally we trained the network on one portion of the Poincaré section and tried to predict another portion of the Poincaré section.

**Figure 8:**
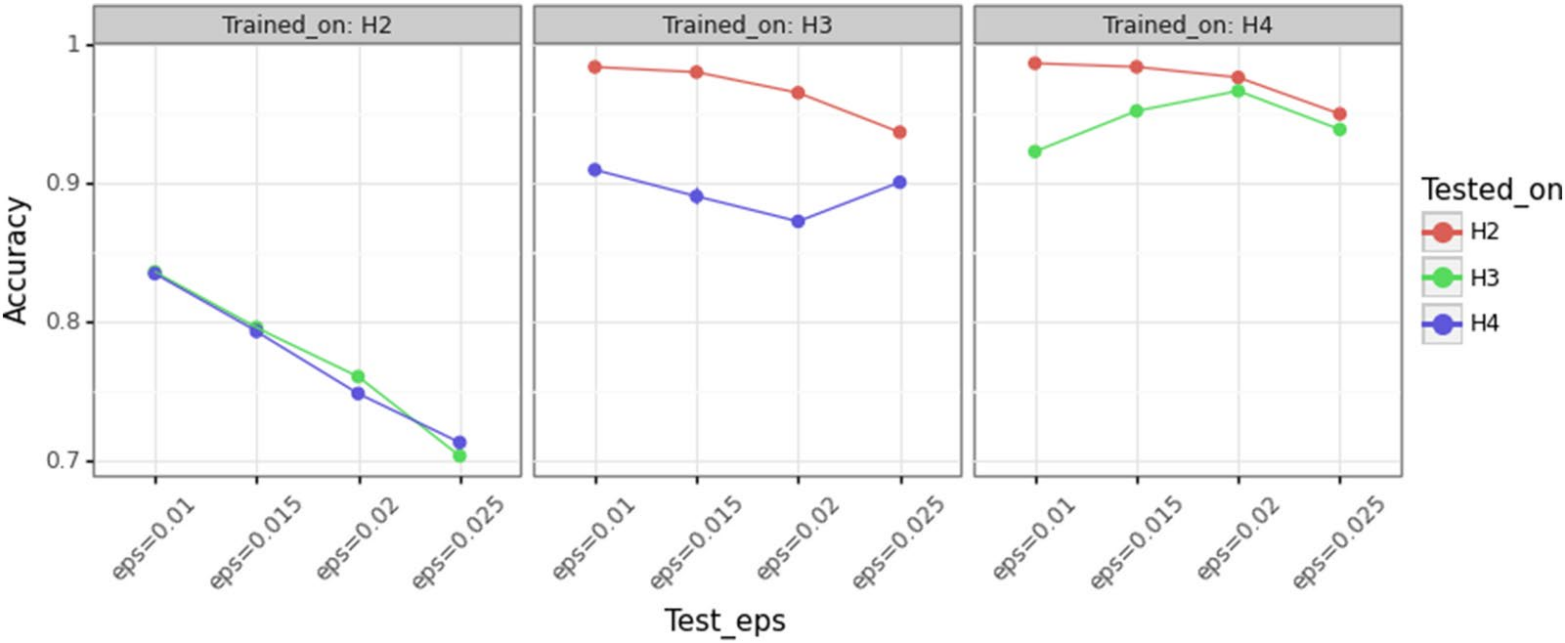
Accuracy comparison among different training-test setups in the three forced pendulum models considered in this work ($H_2, H_3$ and $H_4$). For each of the three panels of the plot, the training set has motions with $\varepsilon = \lbrace 0.01, 0.015, 0.02, 0.025\rbrace$. Each train-test setup (point in the plot) is run 5 times.

In the first set of generalization experiments, we trained InceptionTime on one forced pendulum model and then predicted the index of the time series coming from the other models. For each of the Hamiltonians in $H_2$, $H_3$ and $H_4$, we generated data for four values of $\varepsilon$, namely 0.01, 0.015, 0.02, and 0.025, and trained the network with the data associated to one of the three models. Then, we tested the network performance on data generated by the other two models, for the same values of $\varepsilon$ used in training. This was done 5 times for each case to get an average performance. The results of this experiment are shown in Fig. 8. As it can be seen, training on the simpler model $H_2$ and testing on the other two leads to the worst performance (70% for large values of $\varepsilon$), and instead, training on the model with 4 harmonics $H_4$ manage to generalize well to data from other models (accuracy is above 90%). This indicates that the knowledge learnt from more complex models can be transferred to simpler ones, without the need to train again the network with more data, but the reverse is not working as well.

Another remarkable aspect shown in Fig. 8 is the trend followed by each of the lines, which represents the evolution of the network performance with respect to the values of $\varepsilon$ used to generate the test dataset. Given that the $H_2$ model contains less harmonics than the other two, when the network is trained on time series coming from $H_2$ and tested with time series coming from $H_3$ and $H_4$, there is a clear degradation of the results. On the contrary, when the network is trained on models with a higher number of harmonics it is possible to better characterize the time series generated with simpler models. This is of course even more so when the value of $\varepsilon$ increases as the contribution of the higher harmonics becomes more important. However, there are some cases where the performance of the network improves when increasing the value of $\varepsilon$. As an example, trained on $H_4$, the network learns to predict correctly chaotic motions, but it is not trained to recognize the resonant islands at about $y_0=1.5$ (the resonances corresponding to $1/10\,\cos (2x-3t)$ and $1/5\cos (5x-8t)$ overlap and only for $\varepsilon =0.025$ some small libration islands are visible). Thus, when validating on $H_3$ (green curve in the right panel), we are getting worse results for small values of $\varepsilon$ since in these cases the resonant islands are larger (at $y_0=1.5$, there are the libration islands corresponding to $1/2\cos (2x-3t)$). Conversely, when training on $H_3$ and validating on $H_4$ (blue curve in the middle panel), the accuracy is better for smaller $\varepsilon$ since the number of chaotic motions treated as rotational is smaller.

#### Cartography completion

As mentioned above, we performed a second set of generalization experiments aimed at assessing the ability of the InceptionTime CNN to complete the cartography of a specific dynamical system. We considered two possible ways to complete the cartography: by interpolation and by extrapolation of the training data. Interpolation means that the network is asked to classify unclassified initial conditions that belong to the same range of variability of the initial conditions in the training data set. The data set for the interpolation experiment was composed of 10,201 time series for dynamics (1) with $\varepsilon = 0.01$, and initial conditions in the region $-0.5 \le y_0 \le 1.5$ and $0 \le x_0 < 360$. The 10,201 time series are stored in a long list, in groups of 101 time series where each group corresponds to one value of $y_0$ and values of $x_0$ in the interval [0, 360). Thus the list contains 101 groups of 101 times series, each group ordered according to $y_0$ and each time series in each group ordered according to $x_0$. Out of these 10,201 time series we created a training subset using only one time series every *r* in the list. The other time series were used for testing. Time series were indexed with the convergence method; the network architecture and training configuration is as in the previous sections. Table 1 shows the accuracy for different values of *r*. As it can be seen, the network proves to achieve decent interpolation capabilities (above 90% of accuracy), as long as *r* is lower than 10. This implies that the DL can be used to rapidly increase almost 10 times the resolution of a cartography, or, equivalently reduce the cost by a factor 10.

**Table 1:** Results of the interpolation experiment.

Reduction factor (*r*)	10	9	8	7	6	5	4	3	2
Accuracy	0.063	0.919	0.921	0.923	0.958	0.957	0.965	0.984	0.986

**Figure 9:**
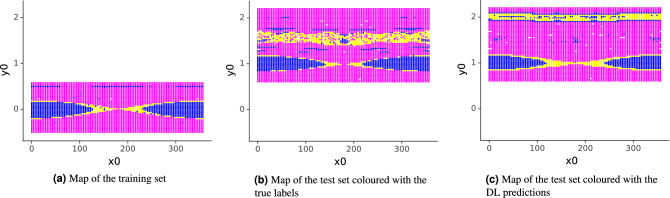
Results of cartography completion (extrapolation experiment). All maps correspond to (3), with $\varepsilon =0.01$.

In the extrapolation experiment, the network is first trained on time series with initial conditions in the range $-0.5 \le y_0 \le 0.6$ and $0 \le x_0 < 360$ and then tested on time series with initial conditions in a region of the cartography different from the one used for training. More specifically, we generated 10,201 time series from (3) with $\varepsilon =0.01$, in the region $-0.5 \le y_0 \le 2.3$. Time series with initial conditions $y_0 \le 0.6$ are used for training (see Fig. 9a), and those with $y_0 > 0.6$ for testing (see Fig. 9b). The result, as average test set accuracy, was $72\%$. As it can be seen in Fig. 9c, the predictions with DL around $y_0=1$ are accurate, while the chaotic band between $y=1$ and $y=2$ is not detected correctly (in fact, it is shifted to higher values of *y*). To explain this, we analyzed side by side time series with similar indexes between the regions $y_0=0$ and $y_0=1$, and saw that the shape of the time series in both regions were similar. Conversely, the motions around $y_0=1.5$ and $y_0=2$ displayed shapes different than those below $y_0\le 0.6$. This suggests that the network is not learning the underlying dynamics of (3), but indexing time series by similitude, and hence, it can extrapolate accurately to regions where the motions have similar time series to those used for training.

### Sensitivity to the number of samples

**Figure 10:**
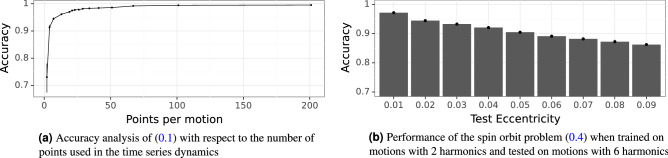
Results of sensitivity to the number of samples and performance in the spin–orbit case.

This section presents the results of an experiment where we assessed the ability of the network to work with reduced time series. To do so, we subsampled each time series in the data set from $H_2$ (see (1)), which has 200 time steps, with an increasing sampling period. We started with a period of 2 (i.e., keeping 1 point every 2), and increased this value up to 128, which resulted in only two points in time. For each (sub)data set, we trained the network 10 times and validated it holding out a random 20% subset of data. Also for this experiment we used the convergence method for indexing and the InceptionTime CNN. The results for a data set of time series from $H_2$ with $\varepsilon =0.01$, $\varepsilon =0.03$ and $\varepsilon =0.05$ are shown in Fig. 10a. As it can be seen, just under 20 time samples per time series are sufficient to achieve an accuracy of about 95%. Moreover, increasing the number of time samples from 100 to 200 brings minimal improvement in accuracy, which is already at  99.5% for 100 samples. However, this experiment is not conclusive because we used problem $H_2$ which has the lowest number of harmonics. More analyses would be required to have proper assessment of the ability of InceptionTime at working with sparse time series.

### Performance in the spin–orbit case

For the more complex case represented by the spin–orbit problem (see Eq. (6)) we used again InceptionTime, with the training configuration as in the previous sections, and the convergence method for indexing. We trained the network on data generated by the 2 harmonics model. To do this, a new synthetic dataset was created with more than 90,000 time series. We took $\epsilon =0.095$, which corresponds to the asteroid *Pallas*, and nine values for the eccentricity, equally spaced from $e=0.01$ to $e=0.09$. For each value, we generate 10000 time series. To analyse the performance of the network, we generate test datasets with the 6-harmonics model in (6), one for each value of the eccentricity used in training.

A total of 5 runs of this experiment were carried out, and the average results are shown in Fig. 7b. As it can be seen, even though it seems that the network generalises correctly from 2 to 6 harmonics, that is just because the motion is fundamentally dictated by the first 2 harmonics. However, as the eccentricity increases, this is less and less the case, and, therefore, the generalisation performance of the network degrades. For higher values of *e*, the coefficients of the harmonics 3–4–5–6 in Eq. (6) get larger and they become of the same order of magnitude of coefficients 1–2. A further analysis of the errors in these experiments showed that, while the chaotic regions were almost perfectly identified, the network misclassified several libration islands as rotational motions. We argue that the higher harmonics affect more libration than rotation, which explains the incorrect identification. The impact of higher harmonics on the characterization of the motion might suggest that the number of samples becomes more important as the number of harmonics increases. On the other hand samples are taken a $t=0 \mod 2\pi$, thus for regular motion with regular sampling we could expect a result similar to Fig. 10a. This is a point that would deserve further investigations in the future to test the possible dependency of the number of samples on the number of harmonics.

Finally, we tested how the network, trained on the spin–orbit case, performed on unseen data from the pendulum case with $\epsilon =0.023$. We obtained $82\%$ of accuracy, which is acceptable considering that the network was trained on another type of system (the spin–orbit model). We leave as future work the study on how to transfer the knowledge from one system (the source) to the other (the target), by fine-tuning the model with just a few indexed time series from the target dynamic model.

## Conclusions and perspectives

In this work we tested the ability of some families of Deep Learning techniques for pattern recognition, to classify types of motion by observing samples taken from time series coming from simple Hamiltonian systems. The DL techniques were trained with different indexing methods based on chaos indicators. We observed that the accuracy of the classification was dependent on the similarity of a time series to the ones used for training, provided that the indexing of the types of motion was accurate. Remarkably we also observed that the DL was able to learn the correct pattern even when the indexing method was producing some incorrect labelling of the time series.

We compared different architectures and demonstrated that, on the test set in this paper, the InceptionTime CNN, had the best performance among the tested architectures. We also demonstrated that by training the CNN on more complex models we could efficiently classify, with good accuracy, the dynamics associated to simpler models. This level of generalization is a promising starting point towards transfer learning in dynamical systems. Our conclusion is that the DL techniques tested in this paper did not learn the dynamics underneath the time series, but rather learnt the key features of the time series; this was sufficient to extrapolate the classification to regions where the trajectories had time series with features similar to those used for the training. As a follow up of this work, it will be interesting to analyze how transfer learning can be used to correctly classify complex problems after training the DL on simpler problems (and viceversa). For example, classifying the spin–orbit problem by progressively adding more harmonic terms. This procedure would make the model more realistic and valid for a wider range of eccentricities. Finally our experiments on the sparsity of the time series suggest that the applicability to real observational data seems to be possible even when data are sparse. This is an interesting direction that deserves further investigations. To be noted that more specific DL algorithms could lead to better results on the data set presented in this paper. However, benchmarking specific DL algorithms on this data set was outside the scope of this paper and will be considered in future work.
